# Introduction: Testing and Refining Marc Lewis’s Critique of the Brain Disease Model of Addiction

**DOI:** 10.1007/s12152-017-9310-2

**Published:** 2017-03-02

**Authors:** Anke Snoek, Steve Matthews

**Affiliations:** 1 Faculty of Health, Medicine & Life Sciences, Maastricht University, Peter Debyeplein 1, 6229 HA Maastricht, The Netherlands; 2 Faculty of Arts, Philosophy Department, Macquarie University, Sydney, NSW 2109 Australia; 3 Centre for Moral Philosophy & Applied Ethics, Plunkett Centre for Ethics, Australian Catholic University (ACU), St Vincent’s Hospital (Plunkett, ACU), 390 Victoria Street, Darlinghurst, NSW 2010 Australia

**Keywords:** BDMA, Disease, Addiction, Lewis

## Abstract

In this introduction we set out some salient themes that will help structure understanding of a complex set of intersecting issues discussed in this special issue on the work of Marc Lewis: (1) conceptual foundations of the disease model, (2) tolerating the disease model given socio-political environments, and (3) A third wave: refining conceptualization of addiction in the light of Lewis’s model.

## Introduction

With the development of neuroimaging techniques in the early nineties, our understanding of addictive behavior advanced significantly. Addiction neuroscience provided evidence for some of the intuitions we held about addiction, posed new questions, and offered the promise of new treatments. Many, like the National Institute on Drug Abuse (NIDA) and erstwhile Director Alan Leshner, thought these neuroscientific insights so revolutionary that they started campaigning for a paradigm shift in how we think about addiction. Their approach to understanding addiction assumed neuroscience as the preeminent level to any investigation of addictive behaviour; famously they claimed, and continue to claim, that addiction is a chronic relapsing brain disease [1].

Prior to this, the so-called moral model of addiction viewed addicts as responsible and blameworthy. On this account addicts have opted for a lifestyle in which they make bad choices, and they are culpable in relation to the actions taken to secure and consume their preferred substance. The brain disease model of addiction (BDMA) was thus welcomed as a much needed alternative to this, but over the years a growing discomfort arose with the exclusive focus on neuroscientific explanations of addictive behaviors. Several theorists were critical of the BDMA [see [2] for an overview], but there had been little *systematic* critique of it, especially from within science.

Then, in 2015, Marc Lewis published *The Biology of Desire. Why Addiction is not a Disease* [2]. This book is unique for several reasons, and arguably the most important of these is that Lewis’s critique derives from within the field: for Lewis, himself, is a developmental neuroscientist who has thought about and published in the addictions field for many years. Lewis offers a reinterpretation of the neuroscientific data on addiction, free from what he calls the disease bias. He criticizes the skewed way in which the neuroscientific data is currently interpreted as showing that a particular human condition is an aspect of neurobiology and therefore not a learned or social problem but an alienating pathology. Lewis criticizes the hierarchy this legitimizes, with medicine dominating at the top and “patients” at the bottom. But, more importantly, when we reframe addiction as the development of coping habits within a social matrix, and when we consider that the brain is designed for exactly that, then the neuroscientific data takes on a completely different meaning. According to Lewis, addiction is an extreme outcome of a normal functioning brain.

Lewis encapsulates his account of addiction this way: it is a habit that grows and self-perpetuates relatively quickly, when we repeatedly pursue the same highly attractive goal. Or, in a phrase, it arises when motivated repetition leads to deep learning. Addictive patterns develop rapidly and become more deeply entrenched than other, less compelling habits, and that is because of the intensity of the attraction that motivates us to repeat them. Quite different conditions may initiate these patterns – based on various intersections of dispositional and environmental factors – but emotional turmoil during childhood or adolescence is typically a culprit, when addictive rewards may serve as a source for relief and comfort.

We took Marc Lewis’s book as a starting point for an extensive analysis of the value of the BDMA, and to explore alternative conceptualizations of addiction. We are extremely grateful to Marc for agreeing to take part in this project for *Neuroethics*, for helping us with suggestions on its direction, for preparing a detailed target article [3], and for his carefully considered responses to the essays. The brief given to our contributors was to consider both the book and target article (and any other related materials from Lewis’s writings), and to address key themes raised by them, especially around the central argument that addiction ought not to be considered a disease, but rather is a condition resulting from the development of deeply ingrained habitual practices. Our invitees include key figures in the field, and are representative of its diversity; we are grateful to them also for their generous efforts for this double issue.

In the remainder of this introduction we set out some salient themes that will help structure understanding of a complex set of intersecting issues: (1) conceptual foundations of the disease model, (2) retaining the disease model given socio-political environments, and (3) A third wave: refining conceptualization of addiction in the light of Lewis’s model.

We approach this via brief introductions of the papers set against these themes.

## The Conceptual Foundations of the Disease Model: Testing Lewis

Many of the papers contain objections to Lewis’s critique of the disease model. And although some of the writers taking this approach also question a range of conceptual foundations presupposed by the disease label, they use Lewis’s account as a launching pad to develop their own positive theories. This approach yielded a range of very interesting new ideas, which we now summarise.

Satel and Lillienfeld [4] point out that before we can decide whether addiction is a brain disease, we must first decide whether it constitutes a disease in the first place. They note that there is very little consensus in psychiatry, and in other domains of medicine, about what constitutes a disease, and they doubt that this issue can be solved scientifically, as Lewis tries, by reinterpreting neuroscientific data (this objection is shared by Wakefield [5], as well as Henden & Gjelsvik [6]). Satel and Lillienfeld think that engaging in this classification debate is potentially fruitless and that the rational response is to develop an understanding of addiction that underscores the way a range of complex behaviours can be analysed in different dimensions ‘…ranging from molecular function and structure and brain physiology to psychology, psychosocial environment, and social and cultural relations.’ The issue of the status of addiction thus cannot avoid questions of normativity that feature in those domains.

Berridge [7] also argues that disease is a tricky concept and one which he tries to avoid using. Like Satel and Lillienfeld he thinks we should ‘put it behind us’ and that we should focus on the actual features and mechanisms of addiction itself. One difficulty he mentions is that ‘brain disease’ brings to mind cases like Alzheimer’s, tumors and strokes, where ‘pathological lesions’ or ‘shriveling neurons’ are present. Addiction is not like that, and it is probably unfair to consider most addicts as significantly brain damaged. However, Berridge take a softer stance than Satel and Lilienfeld, when he says the disease label is not unreasonable and deserves to be tolerated. Distinct neural changes in the brain involved in addiction are extreme enough to be viewed as pathological. He worries about what would happen if those advocating against the brain disease label got their way, warning that ‘they would not like what would follow [for therapy of the affected persons]’. Abandonment of the disease concept might result in a ‘fossilization’ of existing treatment methods. Nevertheless, for the sake of research funding, new and effective treatment methods and public understanding, we need, as Nick Heather puts it, to move to Lewis’s ‘third stage in the governing image of addiction’.

Wakefield [5, 8], who has written extensively about the concept of disease in psychiatry, also takes up the disease classification issue. In a two-part wide-ranging response he argues that even granting the re-interpretation of the brain evidence, Lewis’s arguments against the brain disease classification do not follow. Could it be that Lewis’s story about how development goes in addiction turns out to be compatible with it being a disease (or a disorder) even if we accept the story about the brain changes accompanying addiction? Lewis’s argument had built on the premise that in order for addiction to be a disease, some specific neurological-level mechanism has to be broken. However, when we define disease as being a harmful dysfunction, Wakefield’s key concept here, we see that dysfunction can occur for many reasons other than a broken mechanism. Wakefield argues that although the brains of addicted people are functioning normally, as Lewis outlines, addiction is nonetheless a medical disorder because the desire/deliberation/choice system is not functioning properly. The disorder occurs because of peremptory desires that are operating ‘…outside of biologically designed parameters and [which] override the system’s usual adaptive workings. It is a dysfunction of the brain to be so sensitized to opiates or alcohol as to be unable to function as biologically designed due to need for substance intake, even if this dysfunction occurs via a normal learning system.’

Henden & Gjelsvik take a similar position [6]. Based on the idea of neuroplasticity, Lewis rejects the *neuronormativity* that underlines the BDMA. This is the idea that the brain is a normative thing that can go wrong and be subject to repair. Addiction is a disease, then, according to the BDMA because brain changes deviate from norms of neural function. Lewis’s point, however, is that there is no way to establish these norms. But Henden & Gjelsvik argue that this is only one version of the BDMA. There is another pathway to argue that addiction is a disorder: through dysfunction. Henden and Gjelsvik are torn on the question of whether addiction is a brain disease. Although they think that there are enough good reasons to argue that addiction is a mental or behavioral disorder, they worry about the case for disease notwithstanding their critique of Lewis on this question. For, in the same vein as Wakefield, they think that a deviation from norms of neural function and architecture is neither necessary nor (perhaps) sufficient as ground for disease attribution. As they say, ‘Whether addiction is a brain disease or not is not…something that can be determined solely on the basis of evidence from neuroscience.’

Szalavitz [9] argues as well that the most important thing is that we recognize that addiction is compulsive and has negative consequences, yet she is closely aligned with Lewis to the extent that she agrees addiction is a learning and developmental condition. Szalavitz is taken with Lewis’s analogy between addiction and love, for she thinks that an exploration of attachment neuroscience is a fruitful way to explore the question. The midbrain motivational systems are implicated in bonding, whether it is with offspring or with the continued use of drugs. ‘Disease’, she thinks, is an imprecise word (with a lot of damaging baggage) whose main function is to generate access to medical care. And although she agrees with Lewis about this damaging nature of the disease label she also thinks there are times when people need (evidence-based) medical treatment for their addictive condition. However, she emphasise that addiction as a disease is not like cancer or pneumonia, but rather resembles ADHD or depression. Hence it does not only require medical treatment, but a ‘panoply of social, medical and psychoeducation options.’ Szalavitz, like several of the writers, is concerned not to get bogged down too much in the classification question, but rather to pay attention to the nuance associated with the place of addiction in social settings. Public perceptions of addiction and addicts lead to harm and stigma for example. Another interesting thought she offers is comparison to cases of ADHD, dyslexia and autism. Developmental differences can lead to differences in ability, and disability advocates have argued these are not diseases if they are built out of different wirings in the brain. What we have here is neurodiversity. Addicted persons have a blameless impairment, yet they retain an ability to change.

Flanagan [10] argues that not only is the word ‘disease’ scientifically imprecise, but so is the word ‘addiction’. ‘Disease’ is a folk term and carries too many accretions of competing and inconsistent usages, often with moralistic connotations. So it is not useful. However, in his eyes Lewis and the BDMA seem to agree on the main thing: that addiction is ‘unquestionably destructive’, and that it requires brain changes. The BDMA defines these changes as ‘brain damage’ while Lewis argues that they mistake normal pruning for damage and defines these changes as ‘learning’. Flanagan argues that we shouldn’t aim for a unified theory of addiction, hinting rather that there are many elements on a continuum so that the word ‘addiction’ would be better replaced by ‘substance use disorder’, something that can be assessed along a spectrum from mild to severe. He worries that the attempt to provide pithy short definitions of addiction – as both NIDA and Lewis do – inevitably leaves out much of the phenomena needed for a proper understanding and for making distinctions between what goes on in addiction and what goes on in other conditions captured by the short definitions, the by-catch caught from casting an indiscriminate net. So we need to be more fine-grained in our attempts to get this right.

## Does the Disease Model Deserve to Be Retained Based on Its Socio-Political Merits?

A second theme explored whether the BDMA, although conceptually flawed, deserved to be retained for socio-political purposes in case it has a beneficial effect on treatment and the de-stigmatization of addicted people. The articles of Hall, Carter & Barnett [11], Nagel & Frank [12], and Heather [13] explore these issues. They argue that the BDMA has not fulfilled its promises in improving treatment and reducing the stigma of substance dependent people.

Wayne Hall, Adrian Carter and Anthony Barnett [11]argue for a conceptualisation of addiction ‘that does justice to our understanding of the effects that addictive drugs have on the brain while taking into account evidence that behavioural, social and economic factors also affect drug use and addiction.’ This evidential synthesis however, they argue, has only barely begun. As long as this synthesis has not happened, people will be tempted to use simplified models of addiction like the BDMA.

Nick Heather [13] points out that although several studies have shown that a majority of people have adopted the disease model, this has not led to a decline in moral attitudes towards addiction (see also the paper of Nagel & Frank). Biogenetic explanations of addiction increase endorsement of a new, negative stereotype of substance users in which they are dangerous and incurable. Heather conceptualizes addiction as a disorder of choice. ‘Addiction is seen as a disorder of choice in the sense that it represents a kind of failure to make consistent choices over time…A person makes a strong resolution at time t1 to desist from a specified behaviour at time t2 but, when t2 occurs, fails to carry out that resolution. When that happens repeatedly and distressingly, we can describe this pattern of behavior as addiction.’

Saskia Nagel and Lily Frank [12] address explicitly the question of the relation between addiction and morality. They argue that although one of the alleged benefits of the disease model was that it would de-moralize (and de-stigmatize) addiction, this is not necessarily the case. They explore whether there are non-disease models of addiction that do not have the effect of re-moralizing the condition, concluding that Lewis, among others, offers one. They focus on moral responsibility attributions (via reasons-responsiveness) as the key notion in the moralization question and point out that deficits here derive from multiple causes, disease being just one. They agree that Lewis’s account, among others, can be regarded as compatible with a diminution in reasons-responsiveness.

## A Third Stage in Understanding Addiction: Refining Conceptualization of Addiction in the Light of Lewis’s Model

Many of the contributors critical of Lewis (in some respects), nevertheless find fault with Lewis’s target (the BDMA). For example Satel & Lillienfeld [4] think that Lewis’s account paradoxically reinforces a neurocentric view of addiction because it is written in the language of biology – the lingua franca of human physical disease. And Hall, Carter & Barnett [11] think that Lewis’s analysis of the neurobiological evidence overlaps quite significantly with that of the BDMA. Nevertheless, these authors, and many others, applaud not just Lewis’s attack on the BDMA, but the highly detailed and plausible alternative account he proposes. Moreover, this satisfaction with such an alternative leads to a general sense in which Lewis’s developmental model may be refined in a kind of third wave synthesis. As editors we had a sense of some genuine intellectual progress being made, particularly on the conceptualization question and its implications for practice. We mention here some salient examples.

George Ainslie [14] largely agrees with Lewis’s position. Addiction is the result of a normal process that does not imply lack of responsiveness to motivation. Yet Lewis’s description of addiction in habit terms he thinks does not sufficiently capture the process through which habits become entrenched. Calling addiction a habit is really only the starting point for understanding what it is that persists in this condition. Ainslie describes two recursive phenomena that help explain this. These are dynamic and self-reinforcing phenomena based on ‘intertemporal bargains that have gone bad’ and losses to the hedonic attractions of non-addictive and competing ‘prospects’ in an affected person’s future. He says that when these habit-supporting phenomena interact, the ultimate effect is to ‘hold addiction in place’.

Ted Fenton & Reinout Wiers [15] applaud Lewis on the attempt to undo the arguments of the BDMA, and in particular on his efforts to shine some light on the idea that addiction is an incurable condition and to reject it. Yet, like many other contributors they worry about the severe cases where addiction is very costly. In doing so, they advocate a ‘gradual model’. On this model some cases may be regarded as falling within the disease category, and they take up the question by situating the classification debate within the free will/autonomy debate. In the most severe cases of addiction agency and free will are severely threatened, but in other cases they remain largely intact. The middle range cases combine elements of loss and preserved agency. Thus we need to take on board this variety and take seriously the idea that addiction is on a continuum wherein there are ‘white swans’ (people whose agency is not at all affected by their substance use), ‘black swans’ (people who have barely any agency left due to their addiction), and those in the middle, or ‘grey geese’. This graded perspective takes account of the full range of cases: ‘[i] n extreme cases, with a lot of damage and little or no chance of recovery, the term brain disease may be in place, such as in alcohol-dependent patients suffering from KS [Korsakoff’s Syndrome]. However, in most cases the term appears to be too extreme, and the more developmental dynamic perspective offered by Lewis and others may be more accurate.’

Steve Matthews [16] agrees with many aspects of the account of addiction as habit and learning, but he thinks a nuanced look at addiction is important. Addiction is an elastic concept and it is important to avoid generalizing one’s account of it from some favored range of cases. Most of those affected are not in the grip of a disorder, but there are persuasive reasons to think that some addicted persons are. What are these reasons? Beginning with one of Lewis’s own cases, the case of Johnny (whose addiction to alcohol was extreme), what we see is that the process of deep learning has tipped over into a state of clinically significant impairment and (so) disorder. The argument for this begins by framing addictive processes in terms similar to what goes on in pathological conditions of dissociation, and then to compare the losses we see there to those we see in the extreme cases of addiction. In particular what we see in these cases is a state of ‘mindless’ consumption where desire is absent from the story which explains motivated repetition. That being so we have a case for impairment and disorder if we think also that such impairment is present in the comparison case. Does this mean that addiction is a disorder? Matthews thinks that this question is wrongly posed, because it does not take account of the nuanced nature of cases. He thinks the question also presupposes that addiction is one thing, but, like Snoek, he thinks that the process of addiction contains different phases, and it is only when the affected person reaches that point where their choice-making ability is seriously compromised, and external intervention is required to get well, that addiction deserves to be in the pathological category.

Hanna Pickard [17] agrees with Lewis that it is wrong to pathologize addictive behavior. Rather than providing excuses, we should encourage addicted people to develop a sense of agency and responsibility. Once people acknowledge that their use is a choice, they can change their behavior. Lewis however, rejects the choice model, out of fear that this theory will lead to a moralizing attitude towards addiction. But Pickard argues that we can adopt a choice stance to addiction while not falling into moralization if we distinguish responsibility from blame. Pickard is arguably closest to Lewis on the classification question. She says ‘I agree with Lewis that addiction is not a disease – at least given the typical meaning and implications of that concept. I am also sceptical that, given the state of our current understanding and evidence, we are justified in maintaining that the brain changes caused by repeated drug use are correctly classified as pathological. And I believe Lewis is correct to emphasize the central importance of a sense of agency, empowerment, and personal growth and self-understanding, in overcoming addiction.’

Anke Snoek [18] proposes replacing the notion of addiction as a disease with a notion of a disease-like stage in addiction. She calls this stage the duress stage in addiction, in which the addictive behaviour is largely impervious to the agent’s values and to her available techniques for self-control. Agents in this stage still have a choice, but such choices are under duress. Outlining that this is a *stage* emphasizes that it is not a chronic condition, and can be overcome. However, the addicted agent still needs to develop a wide range of strategies to regain self-control. Snoek raises a question about the relation between a certain conceptualization of addiction and its effect on recovery. Which of the available accounts best bootstraps the agency of the affected person? Although Lewis and Pickard warn that the disease-model of addiction risks making people underestimate their agency, Snoek warns that overestimating people’s agency bears the risk of them becoming demoralized when they relapse, with a consequent loss of belief in self-efficacy. We need a concept of addiction that both acknowledges people’s sense of agency *and* the hardships they encounter in controlling their use. She argues that restoring people’s sense of self-efficacy requires more than just telling them that they can overcome addiction because they do not have a disease. They need to experience success as well in their attempts, and in order to have success they need to be realistic about the hardships they will encounter, and have strategies to overcome the hurdles.

## Concluding Remark

Overall, these articles provide a systematic analysis of the challenges the BDMA faces, the merits and shortcomings of Lewis’s developmental learning model, and many fruitful suggestions for a third wave in how we may conceptualize addiction and the practices surrounding it. It is clear from these articles that we are developing increasingly sophisticated and nuanced theories about the mechanisms that underlie addiction, and as a result new approaches for clinical practices and treatment.
